# Structural (UV) and carotenoid‐based plumage coloration – signals for parental investment?

**DOI:** 10.1002/ece3.2107

**Published:** 2016-04-09

**Authors:** Carsten Lucass, Arne Iserbyt, Marcel Eens, Wendt Müller

**Affiliations:** ^1^ Department of Biology, Behavioural Ecology and Ecophysiology GroupUniversity of AntwerpUniversiteitsplein 12610AntwerpWilrijkBelgium

**Keywords:** Differential allocation, good parent hypothesis, male ornament, parental care, parental investment, sexual selection

## Abstract

Parental care increases parental fitness through improved offspring condition and survival but comes at a cost for the caretaker(s). To increase life‐time fitness, caring parents are, therefore, expected to adjust their reproductive investment to current environmental conditions and parental capacities. The latter is thought to be signaled via ornamental traits of the bearer. We here investigated whether pre‐ and/or posthatching investment of blue tit (*Cyanistes caeruleus*) parents was related to ornamental plumage traits (UV crown coloration and carotenoid‐based plumage coloration) expressed by either the individual itself (i.e. “good parent hypothesis”) or its partner (i.e. “differential allocation hypothesis”). Our results show that neither prehatching (that is clutch size and offspring begging intensity) nor posthatching parental investment (provisioning rate, offspring body condition at fledging) was related to an individual's UV crown coloration or to that of its partner. Similar observations were made for carotenoid‐based plumage coloration, except for a consistent positive relationship between offspring begging intensity and maternal carotenoid‐based plumage coloration. This sex‐specific pattern likely reflects a maternal effect mediated via maternally derived egg substances, given that the relationship persisted when offspring were cross‐fostered. This suggests that females adjust their offspring's phenotype toward own phenotype, which may facilitate in particular mother‐offspring co‐adaptation. Overall, our results contribute to the current state of evidence that structural or pigment‐based plumage coloration of blue tits are inconsistently correlated with central life‐history traits.

## Introduction

Parental care is a widespread behavior within the animal kingdom, because it increases parental fitness through improved offspring condition and survival (Clutton‐Brock 1991; Kölliker et al. 2012). Providing care, however, is costly, for example in terms of time, energy, and a potentially increased predation risk (e.g. Reguera and Gomendio 1999; Milonoff et al. 2004; de Heij et al. 2006), rendering care an investment (Trivers 1974). Thus, parents are expected to trade‐off the amount of care directed toward current offspring against their own future reproductive capacity and survival to maximize lifetime fitness (Stearns 1992).

A factor impinging on these reproductive decisions is partner quality, as this is likely to affect brood value due to direct (e.g. a high level of parental care) and/or indirect (“good genes” for attractiveness or viability, e.g. Kempenaers 2007) benefits for offspring. Thus, it is important for each individual parent to reliably assess the quality of a mate. It has been hypothesized that this can be done based on the expression of (conspicuous) ornamental traits, which are costly to produce or maintain and thus should be honest signals of quality (Zahavi 1975; Hamilton and Zuk 1982; Andersson 1986). These considerations were originally employed to explain conspicuous male ornaments (such as a peacock's tail fan) (Zahavi 1975), but females may also show condition‐dependent phenotypic traits, which may play a role in mate choice and female competition (Amundsen 2000; Kraaijeveld et al. 2007; Clutton‐Brock 2009). Thus, one may expect to find a positive relationship between an individual's ornament and the amount of care it provides (“good parent hypothesis”, Hoelzer 1989; Price et al. 1993). If true, this enables an individual to adjust its investment into current offspring in relation to the quality of its partner (indicating offspring quality) in order to maximize life‐time fitness. More specifically, an individual can increase its investment in current offspring when mated to a high quality partner because of the higher genetic/phenotypic value of the offspring (“differential allocation”, Burley 1986, 1988; Sheldon 2000). However, individuals may also increase investment when mated to a partner, whose traits indicate low quality, thus, compensating via their own investment (“reproductive compensation”, Ratikainen and Kokko 2010). This may be the case when the individual preference is not the same as the general consensus of preference within the species, e.g. when ornaments exploit a sensory bias rather than predicting offspring value (Gowaty 2008).

The signaling function of plumage coloration, in particular UV crown coloration, has been extensively studied in blue tits (*Cyanistes caeruleus*) (reviewed in Parker 2013). Blue tits represent an excellent model system in this context as they provide substantial amounts of biparental care and possess plumage coloration that seems to signal quality. Previous studies have suggested that blue tit UV crown coloration is positively linked to survival (Sheldon et al. 1999; Griffith et al. 2003; Doutrelant et al. 2008), genetic quality (García‐Navas et al. 2009; Ferrer et al. 2015), reproductive success (Parker et al. 2011; Henderson et al. 2013), and sexual attractiveness (Andersson et al. 1998; Hunt et al. 1999). Furthermore, there is evidence that mates alter their investment in function of the UV crown coloration of their partner (Limbourg et al. 2004, 2012, 2013). Females had higher provisioning rates when mated to males with bright UV crown coloration, whereas males provided less food to offspring when mated to bright UV females. This either indicates sex differences in preference or sex differences in reproductive strategies according to UV crown coloration. However, in a recent study males provided less food when paired with an experimentally UV‐reduced female (Mahr et al. 2012). Finally, male UV crown coloration has also been shown to affect prenatal (maternal) investment in terms of yolk androgen deposition (Kingma et al. 2009). Interestingly, yolk androgens have been shown to influence begging behavior (e.g. Schwabl 1996; Eising and Groothuis 2003; Groothuis et al. 2005) and such maternal effects may link postnatal care and UV crown coloration via begging.

However, the role of UV crown coloration as signal of quality in blue tits has been called into question in a recent meta‐analysis (Parker 2013). In fact, Parker (2013) found only evidence for a sex‐difference in plumage coloration (with males reflecting more light in the UV than females) and a weak age‐effect (with birds in their second adult plumage being more intensely colored than birds in their first adult plumage), but no quality trait could be linked to plumage coloration. One of the main conclusions of Parker's review (2013) was to investigate the temporal and spatial consistency and thus significance of blue tit UV crown coloration as a quality signal. Here, we focus on temporal consistency of UV crown coloration in the context of parental care, and additionally investigated these questions for carotenoid based plumage coloration, given the evidence that it may also indicate blue tit quality (Senar et al. 2002; Hidalgo‐Garcia 2006; Doutrelant et al. 2008; García‐Navas et al. 2012; Midamegbe et al. 2013; Ferrer et al. 2015).

In this study, we examined whether parental plumage traits indicate aspects of an individual's quality. We expect that parental investment in the current brood (in terms of clutch size and rate of parental provisioning) is positively linked to parental crown UV chroma and breast plumage carotenoid chroma (“good parent hypothesis”). Parental investment, in turn, should be directly reflected by offspring body condition at fledging (via postnatal care). But also the offspring behavioral phenotype, i.e. begging intensity, may be positively related to the expression of parental ornamental traits, if mothers adjust offspring phenotype to current environmental and social conditions (such as the degree of parental care) via prenatal maternal effects (Mousseau and Fox 1998; Kingma et al. 2009). Lastly, we expect that an individual's investment is not only linked to its own quality but also to that of the partner (“differential allocation hypothesis”, Burley 1986, 1988; Sheldon 2000; and “reproductive compensation hypothesis”, Ratikainen and Kokko 2010). Previous studies on parental investment in relation to the expression of ornamental traits of the partner have revealed opposing strategies between sexes (Limbourg et al. 2013, 2004, 2012; but see Mahr et al. 2012). We performed our study over two consecutive years, which enabled us to study the temporal consistency of any observed pattern. We also cross‐fostered full clutches in 1 year, allowing us to partition pre‐ and postnatal effects, and thus differences in resource allocation at different time points during reproduction.

## Material and Methods

### Study area and general methods

We conducted our experiments in a nest‐box population of blue tits breeding in Peerdsbos, a mature oak‐beech forest near Antwerp (51°16′N, 4°29′E, Belgium) in spring (March – May) 2013 and 2014. By checking nest‐boxes daily we determined clutch size, onset of incubation and hatch date. All clutches of this population were cross‐fostered in 2013 as part of the general experimental procedures on this study population. In 2014, clutches were not cross‐fostered between nests. However, none of our behavioral measures (i.e. begging) appears to be affected by cross‐fostering, so data were pooled (see also Hinde et al. 2009, 2010; Estramil et al. 2013; Lucass et al. 2016b). When clutches were cross‐fostered, full clutches were reciprocally exchanged between two nests of similar clutch size and laying date 3 days prior to the estimated hatch date (see also Lucass et al. 2016a,b).

Day of hatching was defined as day 1. On day 15 we measured tarsus length (to the nearest 0.01 mm, further referred to as offspring size) and body mass (to the nearest 0.01 g) of individual chicks to calculate offspring body condition (by taking the residuals of body mass regressed against tarsus length). Subsequently, each individual was provided with a uniquely numbered metal ring. Parents were caught on day 9 while feeding chicks using nest‐box traps. They obtained a unique color ring combination facilitating further sex identification and we measured their plumage coloration (see below). All experiments were approved by the Ethical Committee for Animal experiments (ECD) of the University of Antwerp (license number 2011‐10).

### Begging behavior

On day 7, the 2nd and 4th chick in a descending weight ranking were individually placed in a warmed artificial nest‐box to record their begging behavior in a food deprivation gradient. We chose the 2nd and 4th chick in order to standardize the begging protocol between broods and thus avoid rank effects on begging (Kilner 1995). Prior to the begging test, chicks were fed with defrosted blue bottle maggots to equalize hunger levels among chicks. We opened the artificial nest‐box (see Estramil et al. 2014 for more information) after 60, 90, and 120 min and videotaped the nestling's begging behavior until it ceased begging, with a video camera (Sony, DCR‐SX 30, Minato, Tokio, Japan). Besides the visual stimulus of a change from darkness to natural daylight, we simultaneously presented an acoustic stimulus, that is a playback of two parental feeding calls, recorded in 2011. Nestlings were immediately returned to their nest after testing.

We analysed the chick's begging behavior from the videotapes, according to a rating scale, modified from Kilner (2002), ranging from 0 (chick is not begging) to 5 (the chick's beak is open, the head is leant back at an angle of 90° and the back of the chick is in a vertical position) (for details see Lucass et al. 2016b,a). For each begging test, scores were applied every second and then summed. For the statistical analysis we used the average values for the begging scores of the two chicks (i.e. the mean of the scores for the two chicks for each of the three measurements taken at 60, 90, and 120 min).

### Provisioning behavior

Between 8 and 9 am in the morning of day 10 we placed an infrared camera (420TVL) underneath the lid of the nest‐boxes, facing downwards into the nest. We discarded the first 30 min of video recordings to avoid a potential influence of this disturbance on our measurements (Kölliker et al. 1998). The following 2 h of the recordings were used for later analysis. Here, provisioning behavior was scored as the number of individual feeding visits per minute (= provisioning rate) using “The Observer XT” software (version 10.0.526, 2010; Noldus Information Technology, Wageningen, The Netherlands).

### Color measurements

When catching adults on day 9 posthatching, we measured coloration of their crown and breast plumage (2013: 28 females and 21 males, 2014: 28 females and 26 males), using a portable Ocean Optics Jaz spectrophotometer with a built‐in pulsed xenon lamp as light source (see Figures S1 and S2 for average reflectance curves of blue tit crown and breast plumage). The spectrophotometer was connected to a bifurcated encased fiber optic probe. Three replicate measures were taken perpendicularly to the feathers relative to a white standard (WS‐1‐SL; Ocean Optics Inc. Dunedin, Florida, USA) and reference measurements were made for each bird. We averaged reflectance curves, covering 320–700 nm, which is the full spectral range a bird can detect (Cuthill et al. 2000; Hart et al. 2000), respectively, for crown and breast plumage of an individual. From this, we calculated crown UV chroma (∑R_320–400_/∑R_320–700_), which represents the purity of UV coloration, and breast plumage carotenoid chroma [(R_700_–R_450_)/R_700_], which represents the relative reflectance around peak absorbance of carotenoids (mainly lutein and zeaxanthin, see Hill 2006). We decided to focus on crown UV chroma and breast plumage carotenoid chroma, as these indices have previously been postulated as important indicators of individual quality in blue tits (Andersson et al. 1998; Hunt et al. 1999; Sheldon et al. 1999; Griffith et al. 2003; Doutrelant et al. 2008; García‐Navas et al. 2009, 2012; Ferrer et al. 2015).

### Statistical analyses

Mixed models were used to test whether individual and partner plumage coloration is predictive for parental investment and offspring phenotype. To explore variability in individual parental provisioning rates, provisioning rates of the partner, (genetic) offspring begging intensity and offspring body condition at fledging, we used brood size at day 10, hatch date (as standardized Julian date), year, parental sex, and plumage coloration (crown UV and breast carotenoid chroma in separate parallel analyses), as well as all two‐way and three‐way interactions with the latter three variables (see Tables 1 and 2) as explanatory variables. Given that we cross‐fostered offspring between nests in 2013 but not in 2014, fledgling condition was analysed once considering the genetic link between parents and offspring (although the latter were raised by foster parents in 2013) and once considering a potential link between offspring and the actual caring parents (i.e. foster parents in 2013, but genetic parents in 2014). We used a generalized linear mixed model with Poisson error distribution and a log link function, to test for effects on clutch size. In this analysis we used the same explanatory variables as above, except that we replaced the hatch date with the lay date of the first egg (again as standardized Julian date). Obviously, brood size was not included in these analyses. All models were adjusted for a bias in statistical independence by including unique nest box number as random effect (provisioning rate) or repeated measures (clutch size and offspring phenotype). Furthermore, five females and three males were measured in two succeeding years. Therefore, all the above analyses were repeated, excluding the data of 2014 for these specific individuals. However, this did not yield different results, hence we considered these successive data points as independent and consistently reported the statistical outcome based on the full dataset. Assumptions for normality were met for all variables (Shapiro‐Wilk: all *W* ≥ 0.92). The outcome of each analysis is reported for the full statistical model, as well as the significant outcome after model reduction. The minimal model was obtained through stepwise backwards elimination by sequentially deleting terms with a *P*‐value higher than 0.05, starting with the least significant interaction. Values of both color indices were standardized within sex and year using z‐scores, which minimizes potential methodological artifacts caused by the use of different spectrophotometers in both years (although units were identical). Sample sizes vary slightly among analyses as we were not able to collect data for all variables at all times. All analyses were performed in SAS 9.3 (SAS Institute Inc., Cary, NC).

**Table 1:** Results of the mixed model approach explaining variation in parental investment and offspring phenotype in relation to crown UV chroma. The table represents the outcome of the full models and the final outcome of reduced models is given in parentheses. Numerator degrees of freedom is 1 in cases b) to e) and df in the table refers to the denominator degrees of freedom. Values for the main effect “Sex” and the interaction “Sex × Year” are not presented in subtable a, b, and e, as those effects are just required for statistical modeling (i.e. the three‐way interaction), but biologically irrelevant. Significant results are indicated in bold

Effect	df	*χ*²/F	*P*
(a) Clutch size			
**Standardized Julian date (lay date 1st egg)**	**1 (1)**	**7.04 (7.29)**	**0.008 (0.007)**
UV chroma	1	1.01	0.315
Year	1	1.37	0.242
UV chroma × Sex	1	1.13	0.288
UV chroma × Year	1	0.62	0.430
UV chroma × Sex × Year	1	0.59	0.444
(b) Begging intensity of genetic offspring			
Standardized Julian date (hatch date)	52	0.25	0.621
Brood size	52	0.44	0.509
UV chroma	36	1.84	0.183
**Year**	**52 (54)**	**6.07 (8.17)**	**0.017 (0.006)**
UV chroma × Sex	36	0.65	0.427
UV chroma × Year	36	1.20	0.282
UV chroma × Sex × Year	36	0.32	0.577
(c) Provisioning rate			
Standardized Julian date (hatch date)	28	2.02	0.166
Brood size	28	0.56	0.459
UV chroma	28	0.02	0.898
Sex	28	0.02	0.880
Year	28	2.42	0.131
UV chroma × Sex	28	0.07	0.795
UV chroma × Year	28	1.25	0.273
Sex × Year	28	0.09	0.764
UV chroma × Sex × Year	28	0.19	0.665
(d) Partner provisioning rate			
Standardized Julian date (hatch date)	27	3.06	0.092
Brood size	27	1.17	0.289
UV chroma	27	0.01	0.915
Sex	27	0.01	0.907
Year	27	5.46	0.027
UV chroma × Sex	27	0.01	0.911
UV chroma × Year	27	0.43	0.519
Sex × Year	27	0.01	0.920
UV chroma × Sex × Year	27	1.87	0.183
(e) Body condition of genetic offspring			
Standardized Julian date (hatch date)	49	0.15	0.700
**Brood size**	**49 (50)**	**4.82 (4.71)**	**0.033 (0.035)**
UV chroma	36	1.66	0.206
**Year**	**49 (50)**	**2.72 (6.29)**	**0.105 (0.015)**
UV chroma × Sex	36	0.19	0.666
UV chroma × Year	36	1.05	0.313
UV chroma × Sex × Year	36	0.10	0.759

**Table 2:** Results of the mixed model approach explaining variation in parental investment and offspring phenotype in relation to breast carotenoid chroma. The table represents the outcome of the full models and the final outcome of reduced models is given in parentheses. Numerator degrees of freedom is 1 in cases b) to e) and df in the table refers to the denominator degrees of freedom. Values for the main effect “Sex” and the interaction “Sex × Year” are not presented in subtable a, b, and e, as those effects are just required for statistical modeling (i.e. the three‐way interaction), but biologically irrelevant. Significant results are indicated in bold

Effect	df	*χ*²/F	*P*
(a) Clutch size			
**Standardized Julian date (lay date 1st egg)**	**1 (1)**	**7.04 (7.29)**	**0.008 (0.007)**
Breast chroma	1	0.68	0.414
Year	1	1.37	0.242
Breast chroma × Sex	1	0.12	0.726
Breast chroma × Year	1	2.50	0.114
Breast chroma × Sex × Year	1	0.43	0.513
(b) Begging intensity of genetic offspring			
Standardized Julian date (hatch date)	52	0.44	0.508
Brood size	52	0.01	0.906
Breast chroma	37 (40)	0.97 (0.49)	0.332 (0.486)
**Year**	**52 (54)**	**7.17 (7.67)**	**0.010 (0.008)**
**Breast chroma** × **Sex**	**37 (40)**	**6.49 (7.48)**	**0.015 (0.009)**
Breast chroma × Year	37	1.86	0.181
Breast chroma × Sex × Year	37	0.14	0.706
(c) Provisioning rate			
Standardized Julian date (hatch date)	29	1.75	0.196
Brood size	29	1.23	0.276
Breast chroma	29	1.41	0.245
Sex	29	0.01	0.913
Year	29	2.48	0.126
Breast chroma × Sex	29	0.71	0.405
Breast chroma × Year	29	0.03	0.858
Sex × Year	29	0.11	0.741
Breast chroma × Sex × Year	29	0.01	0.919
(d) Partner provisioning rate			
Standardized Julian date (hatch date)	29	6.57	0.016
Brood size	29	1.58	0.219
Breast chroma	29	0.80	0.378
Sex	29	0.00	0.983
Year	29	6.19	0.019
Breast chroma × Sex	29	1.89	0.180
Breast chroma × Year	29	0.17	0.679
Sex × Year	29	0.04	0.850
Breast chroma × Sex × Year	29	1.87	0.182
(e) Body condition of genetic offspring			
Standardized Julian date (hatch date)	49	0.25	0.617
**Brood size**	**49 (50)**	**4.59 (4.71)**	**0.037 (0.035)**
Breast chroma	37	0.07	0.794
**Year**	**49 (50)**	**2.51 (6.29)**	**0.120 (0.015)**
Breast chroma × Sex	37	0.00	0.959
Breast chroma × Year	37	0.67	0.418
Breast chroma × Sex × Year	37	1.26	0.269

## Results

**Figure 1:**
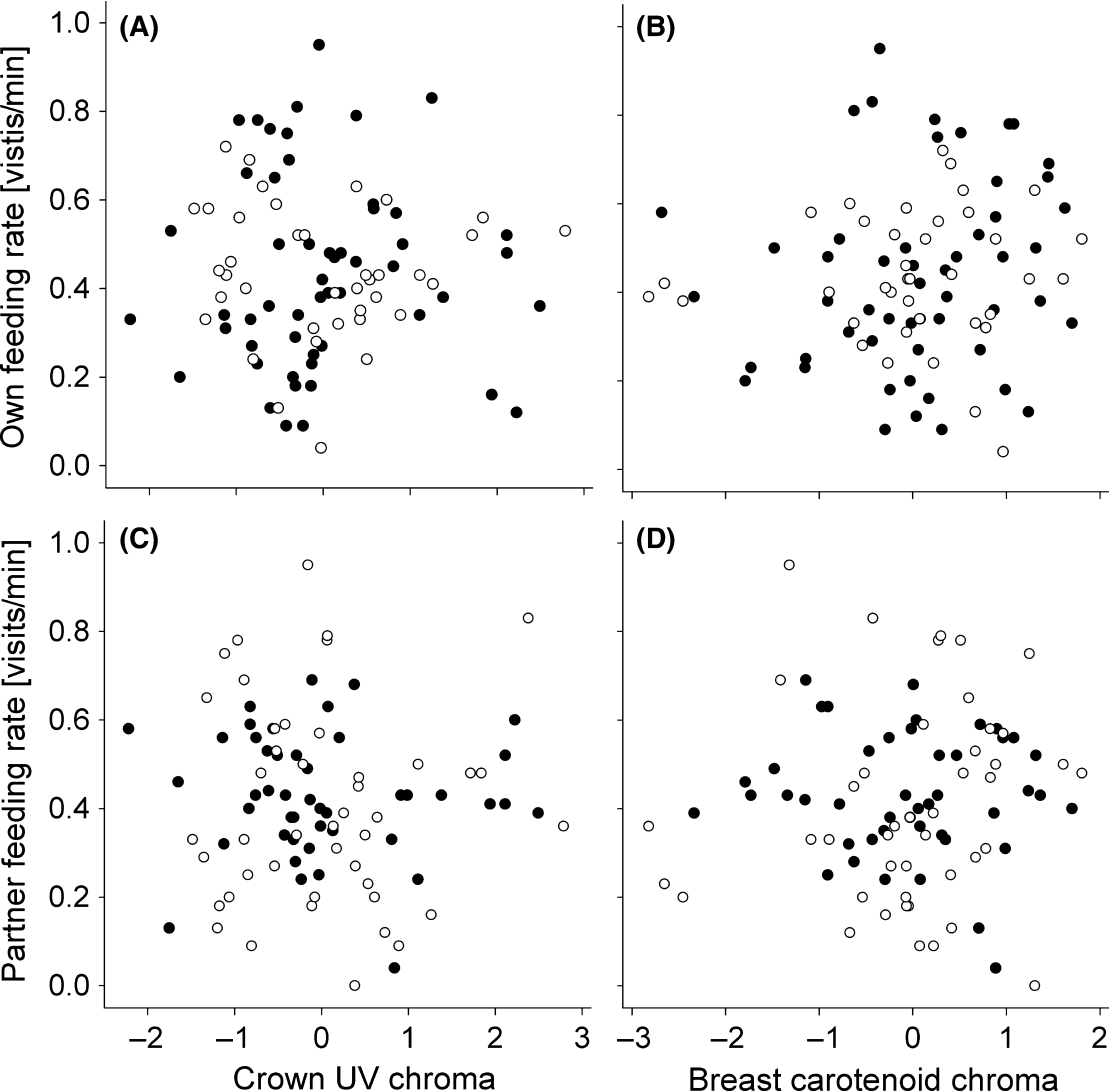
An individual's UV crown coloration (z‐transformed) does not explain variation in own (A), respectively, partner provisioning rate (C). Similarly, an individual's breast carotenoid chroma (z‐transformed) does not explain variation in own (B), respectively, partner provisioning rate (D). Filled circles represent mothers and open circles represent fathers.

The full outcome of the statistical models is presented in Tables 1 and 2. Crown UV chroma never significantly explained variation in parental or partner investment (see Fig. 1A,C), offspring phenotype, or clutch size neither as a main effect, nor in interaction with year, sex or their combination, indicating consistency across years and in sex differences (see Table 1).

**Figure 2:**
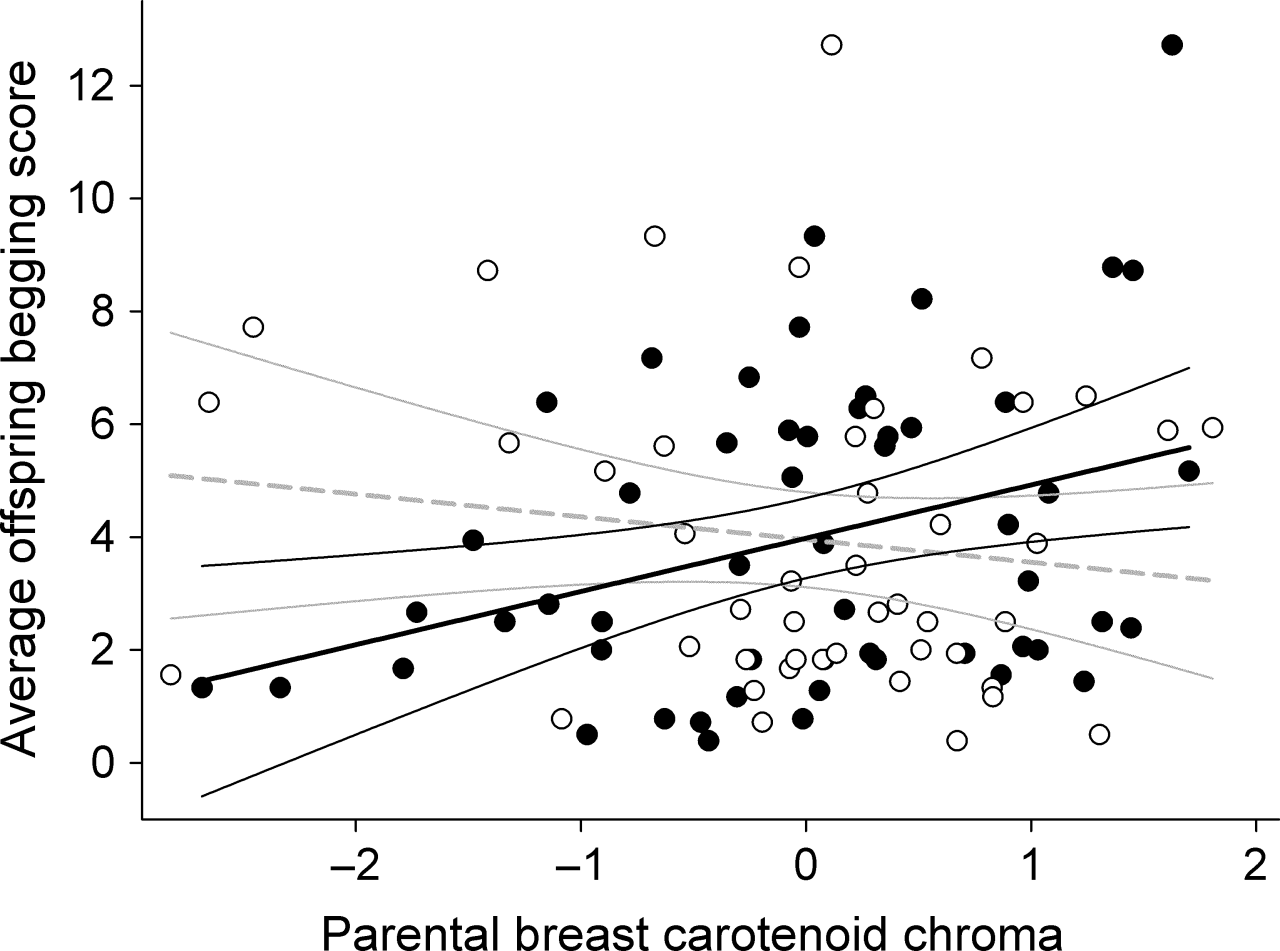
Begging intensity of genetic chicks plotted against parental breast carotenoid chroma (z‐transformed). Filled circles and significant solid black regression line (with 95% confidence bands) represent mothers. Open circles and nonsignificant striped gray regression fit represent fathers.

Breast carotenoid chroma had no significant effect on clutch size (Table 2a), or individual or partner provisioning rates (Table 2c and d, Fig. 1B,D). However, interestingly, parental breast carotenoid chroma affected genetic offspring begging intensity, but this effect differed significantly between sexes (breast chroma × sex interaction; Table 2b; Fig. 2). This pattern was consistent across years (breast chroma × sex × year interaction: *F*_1,37_ = 0.14; *P* = 0.706), and reflects a strong positive relationship in females (Posthoc covariance test for equal slopes: *t*_39_ = 2.56; *P* = 0.014; estimate ± SE = 0.875 ± 0.342; effect size: *r* = 0.146), while there was no such relationship in males (*t*_39_ = −1.37; *P* = 0.179; estimate ± SE = −0.517 ± 0.378; effect size: *r* = 0.094). As we cross‐fostered full clutches between nests in 2013, we also investigated whether the begging intensity of offspring was influenced by breast chroma of individual foster parents in the respective year. However, this was not the case (breast chroma of foster parents × sex interaction: *F*_1,17_ = 0.57; *P* = 0.460).

Investigating whether offspring condition at fledging is linked to plumage coloration of the genetic parents yielded strikingly similar results to the same analysis but with plumage coloration of the actual caring parents (i.e. foster parents in 2013 and genetic parents in 2014) instead of genetic parents. We only report, therefore, on the analysis between fledgling condition and plumage coloration of the genetic parents (see Tables 1e and 2e). Specifically, fledgling condition was only negatively affected by brood size (estimate: −0.039 ± 0.018; effect size: *r* = 0.24) and was higher in 2013 (0.083 ± 0.020) compared to 2014 (−0.100 ± 0.052).

## Discussion

We investigated whether the pre‐ and/or posthatching parental investment of blue tits was related to ornamental plumage traits, expressed by either the individual itself or its partner. In general, most aspects of parental investment were unrelated to plumage coloration, with one exception. Offspring begging intensity was affected by female breast carotenoid coloration in both years. These data contrast a number of previous studies, but confirm the recent view that most previously reported relationships of plumage coloration, in particular of UV crown coloration, with life‐history traits in blue tits are still uncertain (Parker 2013).

### UV crown coloration

We found that an individual's UV crown coloration was not predictive for its level of parental investment neither pre‐ nor posthatching. Prehatching investment was measured here in terms of clutch size and begging behavior, with begging reflecting among others maternal effects such as maternally derived yolk androgens (e.g. Schwabl 1996; Eising and Groothuis 2003; reviewed in Groothuis et al. 2005). But despite the fact that prehatching investment is strongly dependent on females (Mousseau and Fox 1998), no relationships with female UV crown coloration were found. Maternal resource allocation was also unaffected by the UV crown coloration of its partner. The lack of an effect of partner coloration on clutch size contrasts with a previous meta‐analysis dissecting the evidence for differential allocation (Horváthová et al. 2012). This meta‐analysis revealed that females of biparental species have larger clutches when exposed to (or paired with) attractive males.

Furthermore, it has been shown that blue tit mothers modulate egg yolk androgen concentrations in relation to male UV crown coloration (Kingma et al. 2009; see also Table 1 in the same paper for an overview of relationships in other species), which in turn should lead to changes in offspring phenotype. However, it may be that changes in terms of yolk androgens are too limited to become functionally significant, which could explain why we did not find that begging intensity varies with male UV crown coloration. Alternatively and also potentially more likely given the clutch size data, there is no relationship between male attractiveness, here UV crown coloration, and female prehatching investment, at least not in our study.

Similar patterns were found for parental investment posthatching, measured in terms of parental provisioning rates, which were unrelated to UV crown coloration of the focal individual. This (absent relationship) corresponds with the observation that offspring body condition at fledging was unrelated to UV crown coloration. Body condition at fledging can be interpreted as an integrative measure of provisioning over the entire nestling period, it is, however, impossible to unravel the different contributions from the sexes. These results are also in line with a study by Limbourg et al. (2012) on the same species. However, Limbourg et al. (2004, 2012, 2013) found convincing evidence, both in correlative and experimental studies, that females increase provisioning with increasing male UV crown coloration, whereas males decreased provisioning with increasing female UV. We, however, found that provisioning is not adjusted toward UV crown coloration of the partner, although our study is in fact almost identical with respect to the set‐up of the correlative study by Limbourg et al. (2012), which was only performed in a different study population in different years and we did not distinguish the age of the parents (yearling or older). The results of our study, together with a recent experimental study in yet another blue tit population (Mahr et al. 2012), revealing a pattern that contrasts with the one reported by Limbourg et al. (2012, 2013), indicate thus a high level of inconsistency on the spatial level.

### Breast carotenoid coloration

Carotenoid‐dependent plumage traits in blue tits have received considerably less attention compared to other species. For example, house‐finch (*Carpodacus mexicanus*) females preferentially mate with males that display carotenoid‐based bright red plumage (Hill 1990), as these males have a higher overwinter survival (Hill 1991), are in better nutritional condition (Hill and Montgomerie 1994) and they feed the incubating female more than pale yellow males do (Hill 1991). But also in blue tits recent experimental and correlative evidence indicates that carotenoid‐based coloration may act as a signal, reflecting individual quality (Senar et al. 2002; Hidalgo‐Garcia 2006; Doutrelant et al. 2008; García‐Navas et al. 2012; Midamegbe et al. 2013; Ferrer et al. 2015).

We found that an individual's breast carotenoid coloration was unrelated to clutch size, but we found a consistent positive association between offspring begging intensity and their mother's carotenoid coloration. This intriguing finding likely reflects a maternal effect, potentially mediated via yolk androgens, given that the relationship between offspring begging intensity and maternal breast carotenoid chroma is consistent between years, although offspring were cross‐fostered in 1 year (2013), and the fact that yolk androgens are known to have long‐lasting effects on the phenotype (Eising and Groothuis 2003; Groothuis et al. 2005; Müller et al. 2007). It has been previously shown that carotenoid‐supplemented mothers lay eggs with higher carotenoid concentrations (e.g. Blount et al. 2002a,b; Bortolotti et al. 2003; Biard et al. 2005), which may result in more intense begging offspring (Helfenstein et al. 2008). If females adjust their offspring's phenotype toward her own phenotype, it may facilitate in particular mother‐offspring co‐adaptation, as has been previously found (see Kölliker et al. 2000).

We then focused on the relationship between prehatching maternal investment and partner carotenoid plumage coloration. Such a relationship had been previously shown, for example, for blue‐footed booby mothers that adjusted their prehatching reproductive investment to a carotenoid‐based male trait (Velando et al. 2006), a pattern, that we could not confirm in blue tits. However, the trait measured in the latter study (foot skin color) varies rapidly with nutritional status (Velando et al. 2006), which is in contrast with a rather static plumage trait as measured in this study (see below for a more extensive discussion on the meaning of signals in feathers). Being a static trait may be one explanation as to why male carotenoid‐based plumage coloration did not affect maternal investment prehatching.

However, females did not adjust their prehatching investment in relation to male breast carotenoid coloration. This likely related to our observation that breast carotenoid coloration is not predictive for provisioning or offspring body condition at fledging.

### Plumage coloration and parental investment in blue tits

Initially, accumulated evidence pointed toward blue tit plumage coloration acting as a signal of individual quality (Andersson et al. 1998; Hunt et al. 1999; Sheldon et al. 1999; Griffith et al. 2003; Doutrelant et al. 2008; García‐Navas et al. 2009, 2012; Henderson et al. 2013; Ferrer et al. 2015). However, a recent meta‐analysis revealed that our gain in knowledge even after more than 10 years of studying functional aspects of in this case UV crown coloration is particular limited (Parker 2013). By the time of this review the relationship of UV crown coloration and parental care had not been published, while these studies showed a high level of consistency across years within a population in The Netherlands (Limbourg et al. 2004, 2012, 2013). We therefore set out to test the robustness of these findings, implementing the temporal (by investigating 2 years) consistency of aspects in our study. As advocated among others by Parker (2013), research benefits from studies that focus on within‐population consistency of previously reported patterns by replicating. Having detected (in)consistencies between populations may on one hand reflect (the lack of) an overall relationship. Or it may, on other hand, stimulate studies investigating those ecological and/or social factors that drive such temporal and spatial variation.

One important aspect that should always be kept in mind is that the expression of (pigment‐based) plumage coloration is determined at molt (which takes place between July and September, Cramp and Perrins 1993) and, therefore, most strongly reflects an individual's quality in that (relatively short time) period (Hill 2006). That is to say that carotenoid‐based plumage critically depends on the amount of ingested carotenoids during molt (Saks et al. 2003). But we currently lack knowledge on whether carotenoids are actually limited during that period or not (Olson and Owens 1998), a central aspect for its signaling function.

As UV crown coloration depends on the nano‐structural arrangements of feathers (Shawkey et al. 2003), its expression may again depend on an individual's condition during molt. But numerous other processes will impinge on its expression before mating/caring for offspring, such as feather abrasion, bleaching and accumulation of dirt (Figuerola and Senar 2005; Delhey et al. 2006). These processes are thought to be responsible for the changes in UV crown coloration, that have been observed with the time of the season (Figuerola and Senar 2005; Delhey et al. 2006), and are likely to introduce additional noise on the signal. Thus, it remains to be shown how individual and territory quality at molt relate to the ability to forage caterpillars (the main diet of dependent nestlings) and territory quality during the breeding season, also given the high level of stochasticity in for example environmental conditions.

## Conclusions

We investigated the relationship between blue tit plumage coloration and parental investment – stimulated by previously reported intriguing patterns of parental investment adjusted to (changes in) partner UV crown coloration. However, our results do not confirm that individual investment of blue tits into current offspring varies with plumage coloration, neither of the individual itself nor of its partner. An exception to this is maternal breast carotenoid coloration that was positively linked to offspring begging intensity, likely reflecting a maternal effect. Thus, observed patterns of investment in relation to partner plumage coloration appear to be less consistent than previously thought, at least across years and populations. This study adds to the uncertainty of the signaling function of UV crown coloration in blue tits, but potentially also in other bird species. Furthermore, our results suggest that such inconsistency could also apply for relationships with carotenoid‐based plumage coloration.

## Conflict of Interest

None declared.

## Supporting information


**Figure S1.** Mean reflectance curves of crown plumage of male (*N* = 47) and female (*N* = 56) blue tits.
**Figure S2.** Mean reflectance curves of carotenoid based breast plumage of male (*N* = 47) and female (*N* = 56) blue tits.
