# Supplementary material for: Structural (UV) and carotenoid‐based plumage coloration – signals for parental investment?
Source: Ecol Evol. 2016 Apr 9;6(10):3269–79. doi: 10.1002/ece3.2107 (PMC4870211; doi:10.1002/ece3.2107)
Supplement: Supplementary file 1 — Figure S1. Mean reflectance curves of crown plumage of male (N = 47) and female (N = 56) blue tits. Figure S2. Mean reflectance curves of carotenoid based breast plumage of male (N = 47) and female (N = 56) blue tits. [file ECE3-6-3269-s001.pdf]

Supporting Information

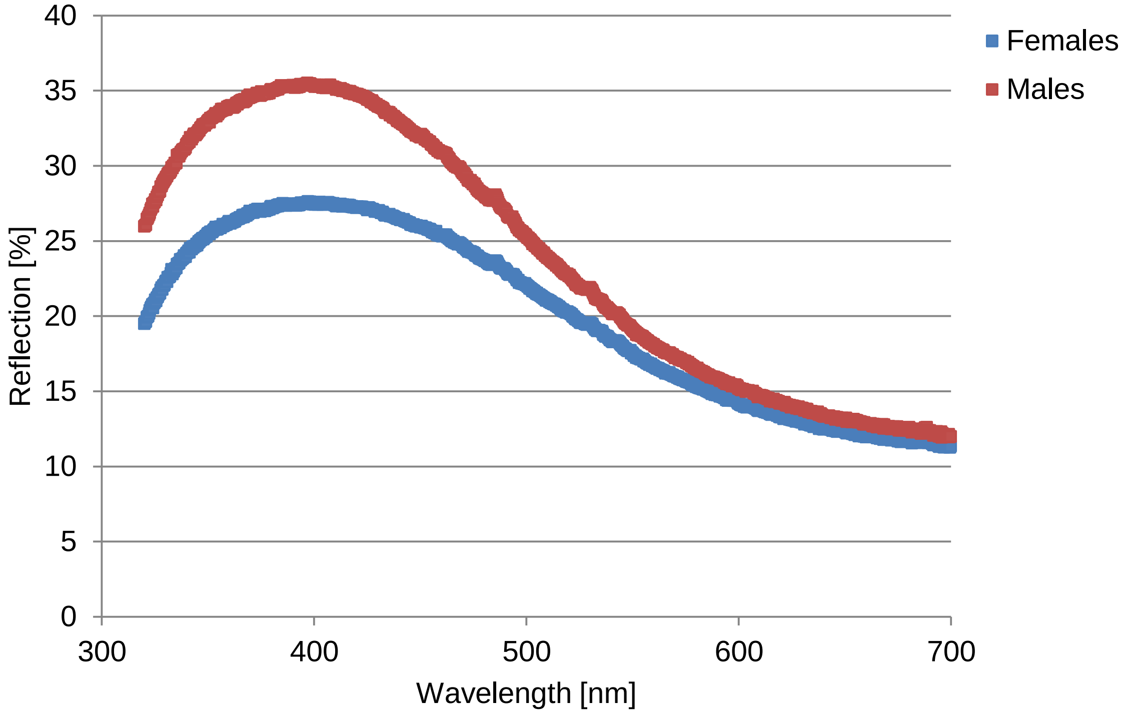

Figure S1. Mean reflectance curves of crown plumage of male (N=47) and female (N=56) blue tits. Data were collected during nestling provisioning (day 9 post-hatching) in two different breeding seasons (2013 and 2014) in a nest-box breeding blue tit population (Peerdsbos, Belgium).

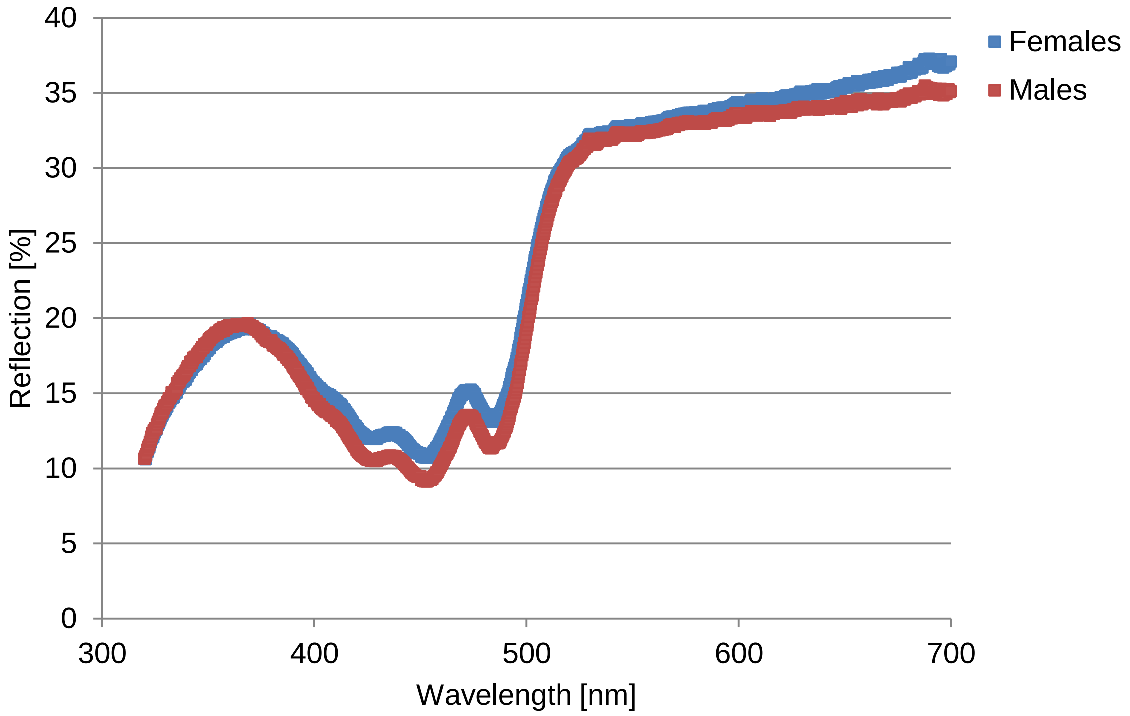

Figure S2. Mean reflectance curves of carotenoid based breast plumage of male (N=47) and female (N=56) blue tits. Data were collected during nestling provisioning (day 9 post-hatching) in two different breeding seasons (2013 and 2014) in a nest-box breeding blue tit population (Peerdsbos, Belgium).
